# Pediatric vascular compression of the esophagus: Endoluminal functional lumen imaging probe as a complement to imaging and endoscopy

**DOI:** 10.1002/jpr3.70140

**Published:** 2026-01-14

**Authors:** Brett J. Hoskins, Paroma Bose, Ryan T. Pitman

**Affiliations:** ^1^ Division of Pediatric Gastroenterology, Hepatology, and Nutrition, Department of Pediatrics Indiana University School of Medicine, Riley Hospital for Children at IU Health Indianapolis Indiana USA

**Keywords:** aberrant subclavian artery, compliance, distensibility index, dysphagia lusoria, vascular anomalies

## Abstract

**Objectives:**

Vascular anomalies can cause extrinsic esophageal compression, leading to dysphagia or feeding difficulties in children. Diagnosis typically relies on imaging and endoscopy, which may under‐ or overestimate functional narrowing. Endoluminal functional lumen imaging probe (EndoFLIP) provides luminal parameters in real‐time, but its role in vascular compression is not well‐defined.

**Methods:**

We retrospectively reviewed children with vascular anomalies who underwent upper endoscopy with EndoFLIP (July 2021–April 2025). Demographics, imaging, endoscopic/EndoFLIP findings, and outcomes were recorded. In select cases, paired measurements at the lower esophageal sphincter (LES) and compression site were compared at matched balloon volumes.

**Results:**

Eight patients (mean age 12.1 years; range 6–17; 5 females) were included. Vascular anomalies included left aortic arch with aberrant right subclavian artery (*n* = 5), double aortic arch (*n* = 2), and right aortic arch with anomalous left subclavian artery (*n* = 1). EndoFLIP detected narrowing in 6/8 (75%), compared with 4/8 (50%) on endoscopy and 6/7 (86%) on upper gastrointestinal (UGI) series. Two patients had narrowing on EndoFLIP despite normal endoscopy; two others had UGI narrowing but normal EndoFLIP and endoscopy. Paired measurements (*n* = 2) showed markedly reduced diameter and distensibility at the compression site versus the LES. Management included surgical repair (*n* = 3), dietary modification (*n* = 2), and proton‐pump inhibitor therapy (*n* = 3), with variable symptom improvement. No procedural complications occurred.

**Conclusions:**

EndoFLIP was safe, feasible, and provided meaningful complementary physiologic information in this heterogeneous cohort. These preliminary findings support a potential diagnostic role for EndoFLIP in esophageal vascular compression, warranting confirmation in larger, standardized, prospective studies.

## Abstract (highlights)

**What is Known:**

• Vascular anomalies can cause extrinsic esophageal compression, leading to dysphagia in children.
• Diagnosis traditionally relies on imaging and endoscopy, which may under‐ or overestimate functional narrowing.
• Endoluminal functional lumen imaging probe (EndoFLIP) provides real‐time luminal diameter and distensibility measurements but has limited pediatric data in vascular compression.

**What is New:**

• EndoFLIP was safe and feasible in children with vascular anomalies.
• EndoFLIP identified subtle functional narrowing not appreciated on endoscopy and helped exclude clinically significant compression when symptoms were attributable to other causes.
• Paired measurements demonstrated reduced distensibility at the compression site compared with the lower esophageal sphincter (LES) in select patients, suggesting potential physiologic relevance.
• These preliminary findings highlight a possible diagnostic role for EndoFLIP in evaluating esophageal vascular compression, warranting further validation in larger, standardized, prospective studies.

## 1 INTRODUCTION

Vascular anomalies of the aortic arch and great vessels are an uncommon yet clinically important cause of extrinsic esophageal compression in children. Presentations may include dysphagia, feeding difficulties, aspiration, and weight loss,^1^ and in some cases are identified incidentally on imaging in otherwise asymptomatic children. When due to esophageal compression from an aberrant right subclavian artery (ARSA), the condition is classically termed dysphagia lusoria. Common variants include left aortic arch with ARSA, double aortic arch (DAA), and right aortic arch with anomalous left subclavian artery (ALSA).^2^, ^3^ While ARSA is found in up to 2% of the general population, symptomatic cases in pediatrics are less frequent. Because vascular rings such as DAA typically cause more fixed, circumferential compression than ARSA, which exerts oblique or posterior indentation, symptom severity and functional impact differ across subtypes.

Diagnosis is traditionally made using imaging—fluoroscopic upper gastrointestinal (UGI) series to assess esophageal contour, and cross‐sectional modalities such as computed tomography (CT) angiography, or magnetic resonance imaging (MRI) to delineate vascular anatomy and its relationship to the esophagus.^4^, ^5^ However, these modalities cannot directly assess the functional impact of compression on luminal caliber. Endoscopic visualization can identify mucosal indentation or narrowing but is subjective and may not reflect the true degree of functional impairment.

Endoluminal functional lumen imaging probe (EndoFLIP; Medtronic, Minneapolis, MN) uses impedance planimetry to provide objective, real‐time measurements of luminal parameters including diameter and distensibility index (DI). In pediatrics, EndoFLIP has been primarily applied to esophageal motility disorders, pre‐ or post‐intervention assessment, and conditions such as eosinophilic esophagitis.^6^, ^7^, ^8^ Literature on its use for pediatric vascular compression is minimal.^9^ This pilot study describes our institutional experience and explores EndoFLIP's potential role as a complementary diagnostic tool in children with suspected vascular esophageal compression.

## 2 METHODS

### 2.1 Ethics statement

This study was approved by the Indiana University Institutional Review Board (Protocol #27494). A waiver of informed consent was granted due to the retrospective design.

### 2.2 Study design

A retrospective review was conducted of patients aged 0–18 years with known vascular anomalies who underwent upper endoscopy with esophageal EndoFLIP at Riley Hospital for Children between July 2021 and April 2025. No patients were excluded from the analysis. For each patient, demographic data, vascular anatomy, imaging findings, endoscopic observations, EndoFLIP measurements, mucosal biopsy results, treatment approach, and follow‐up outcomes were recorded. Because the cohort was small and heterogeneous, with variable vascular anatomies and symptom severity, EndoFLIP findings were analyzed descriptively rather than compared quantitatively across subtypes. Imaging was reviewed for luminal narrowing or esophageal indentation. Endoscopic narrowing was defined as visible indentation, luminal reduction, or mucosal puckering. EndoFLIP measurements were obtained at the lower esophageal sphincter (LES) and suspected compression site, as confirmed endoscopically. DI was calculated as cross‐sectional area divided by intra‐balloon pressure (mm^2^/mmHg).

In two patients, paired EndoFLIP measurements at the site of compression and the LES were analyzed for qualitative comparison. Formal statistical testing between LES and compression sites was not performed, as only two patients had paired measurements. The LES served as an internal control site since it is routinely evaluated during EndoFLIP procedures, readily available in retrospective review, and exhibits reproducible distensibility properties—providing a consistent within‐patient reference. EndoFLIP data from the esophageal body were not consistently available, precluding standardized comparison to non‐compressed regions.

### 2.3 Procedural standardization

All procedures were performed under general anesthesia using either the EF‐322N or EF‐325N catheters (Medtronic; 16 cm or 8 cm measurement segment, respectively), chosen according to patient height. EndoFLIP was performed after diagnostic endoscopy, under total intravenous anesthesia using propofol and/or fentanyl. Sevoflurane was not used in any case. Balloon volumes were increased in 10 mL increments (range 20–60 mL) until adequate esophageal distension or a plateau in pressure response was reached. Not all distension volumes were attainable in smaller patients, resulting in variation across cases. Differences in inflation volume between the LES and compression site reflected anatomic constraints and patient size, as overdistension risk limited maximum inflation at proximal levels. When EndoFLIP did not demonstrate luminal narrowing, site‐specific measurements were not repeated, contributing to procedural variation and reducing direct comparability across sites.

## 3 RESULTS

### 3.1 Demographics

Eight patients (mean age 12.1 years; range 6–17; 5 females) met inclusion criteria (Table 1). Diagnoses included ARSA (*n* = 5; one with pre‐ and postreconstruction data), DAA (*n* = 2; one postreconstruction), and right aortic arch with ALSA (*n* = 1). All presented with dysphagia, with three also having feeding refusal and two experiencing weight loss.

**Table 1:** Patient characteristics, diagnostic findings, and outcomes.

Patient	Age (years)	Gender	Race/ethnicity	Vascular anomaly	Chief complaint	UGI narrowing	Endoscopy narrowing	EndoFLIP narrowing	Biopsies	Complications	Treatment	Outcome
1	12	Female	White	Left aortic arch with ARSA	Dysphagia	Yes	Yes	Yes	Normal	None	Surgical repair	Partial improvement
2	12	Male	White	Left aortic arch with ARSA	Dysphagia	Yes	No	No	Normal	None	PPI	Resolution
3	11	Female	White	DAA s/p surgery	Dysphagia	Yes	Yes	Yes	Normal	None	Surgical repair	Resolution
4	6	Female	Black	Left aortic arch with ARSA	Dysphagia	NC	Yes	Yes	Normal	None	Planned surgical repair	Pending surgery
5	17	Male	White	Right aortic arch with ALSA	Dysphagia	No	No	Yes	Normal	None	Dietary changes	Partial improvement
6	16	Female	White	Left aortic arch with ARSA	Dysphagia	Yes	No	No	Normal	None	PPI	Resolution
7	6	Female	White	DAA s/p surgery	Dysphagia	Yes	No	Yes	Normal	None	Dietary changes	Partial improvement
8	17	Male	White	Left aortic arch with ARSA	Dysphagia	Yes	Yes	Yes	48 eosinophils per HPF	None	PPI	Resolution

Abbreviations: ALSA, anomalous left subclavian artery; ARSA, aberrant right subclavian artery; DAA, double aortic arch; EndoFLIP, endoluminal functional lumen imaging probe; HPF, high‐power field; NC, not completed; PPI, proton pump inhibitor; s/p, status post; UGI, upper gastrointestinal.

### 3.2 Diagnostic findings

EndoFLIP detected esophageal narrowing in 6/8 patients (75%) compared with 4/8 (50%) on endoscopy and 6/7 (86%) on UGI imaging (Table 2). One patient (Patient 4) did not undergo UGI imaging. Two patients (Patients 5 and 7) had narrowing on EndoFLIP despite normal esophageal caliber reported on endoscopy. In Patient 5, the vascular anomaly was initially identified on echocardiography and subsequently confirmed on chest CT, both prior to gastrointestinal evaluation and endoscopy. In Patient 7, EndoFLIP showed a mildly reduced luminal diameter with a normal DI at the prior compression site (postsurgery), with preserved distensibility at the prior compression site. Two others (Patients 2 and 6) had narrowing on UGI imaging but normal EndoFLIP and endoscopy. No patients had narrowing on endoscopy without corresponding EndoFLIP narrowing.

**Table 2:** EndoFLIP measurements at the lower esophageal sphincter and site of vascular compression.

Lower esophageal sphincter						
Patient	Inflation (mL)	Diameter (mm)	DI (mm²/mmHg)	CSA (mm²)	Pressure (mmHg)	Planimetry findings
1	40	11.0	4.2	93	22	Diminished contractility*
	60	13.0	5.0	159	32	
2	40	12.0	9.9	183	19	RACs present
	50	10.5	4.3	118	28	
3	30	14.0	9.0	144	16	RACs present
	40	20.0	10.0	300	30	
4	20	7.3	1.2	42	34	RACs present
	25	9.8	1.8	59	38	
	30	20.0	6.0	255	43	
5	40	8.6	1.7	NA	NA	RACs present
	50	13.0	3.9	NA	NA	
6	40	10.6	3.9	89	23	RACs present
	50	12.5	4.4	123	28	
	60	14.3	5.0	161	32	
7 (Postsurgery)	20	11.7	5.3	108	20	RACs present
	30	12.3	6.6	129	20	
8	30	6.2	1.6	30	18	RACs present
	40	11.7	3.6	107	29	
	50	17.0	6.7	227	34	

**Site of compression**					
**Patient**	**Inflation (mL)**	**Diameter (mm)**	**DI (mm²/mmHg)**	**CSA (mm²)**	**Pressure (mmHg)**
1	40	11.0	4.7	104	22
	60	10.0	4.3	106	59
2	NA	NA	NA	NA	NA
3	30	4.8	1.2	19	16
	40	7.5	1.7	51	30
	50	10.0	2.2	88	40
	60	12.0	2.5	138	55
4	10	8.5	2.2	57	26
	15	8.9	1.9	62	33
	30	10.3	2.4	88	36
5	40	12.0	NR	NR	NR
6	NA	NA	NA	NA	NA
7 (Postsurgery)	20	13.0	6.0	120	20
8	NR	NR	NR	NR	NR

*Fewer than six secondary contractions per minute or complete absence of contractions.

Abbreviations: CSA, cross‐sectional area; DI, distensibility index; EndoFLIP, endoluminal functional lumen imaging probe; NA, not applicable; NR, not recorded; RACs, repetitive anterograde contractions.

During EndoFLIP assessment, most observed narrowing appeared fixed during sustained distension, although subtle pulsatility was occasionally visible, consistent with dynamic vascular motion. Repetitive anterograde contractions (RACs), defined as regular, propagated secondary contractions occurring in response to sustained esophageal distension, were present in 7/8 patients (88%). One patient (Patient 1) had diminished contractility of unclear etiology, defined as fewer than 6 secondary contractions per minute or complete absence of contractions. High‐resolution manometry was planned for Patient 1 but had not yet been completed at the time of review. Biopsies were normal in 7/8 cases (88%) and one showed eosinophilic esophagitis.

**Figure 1:**
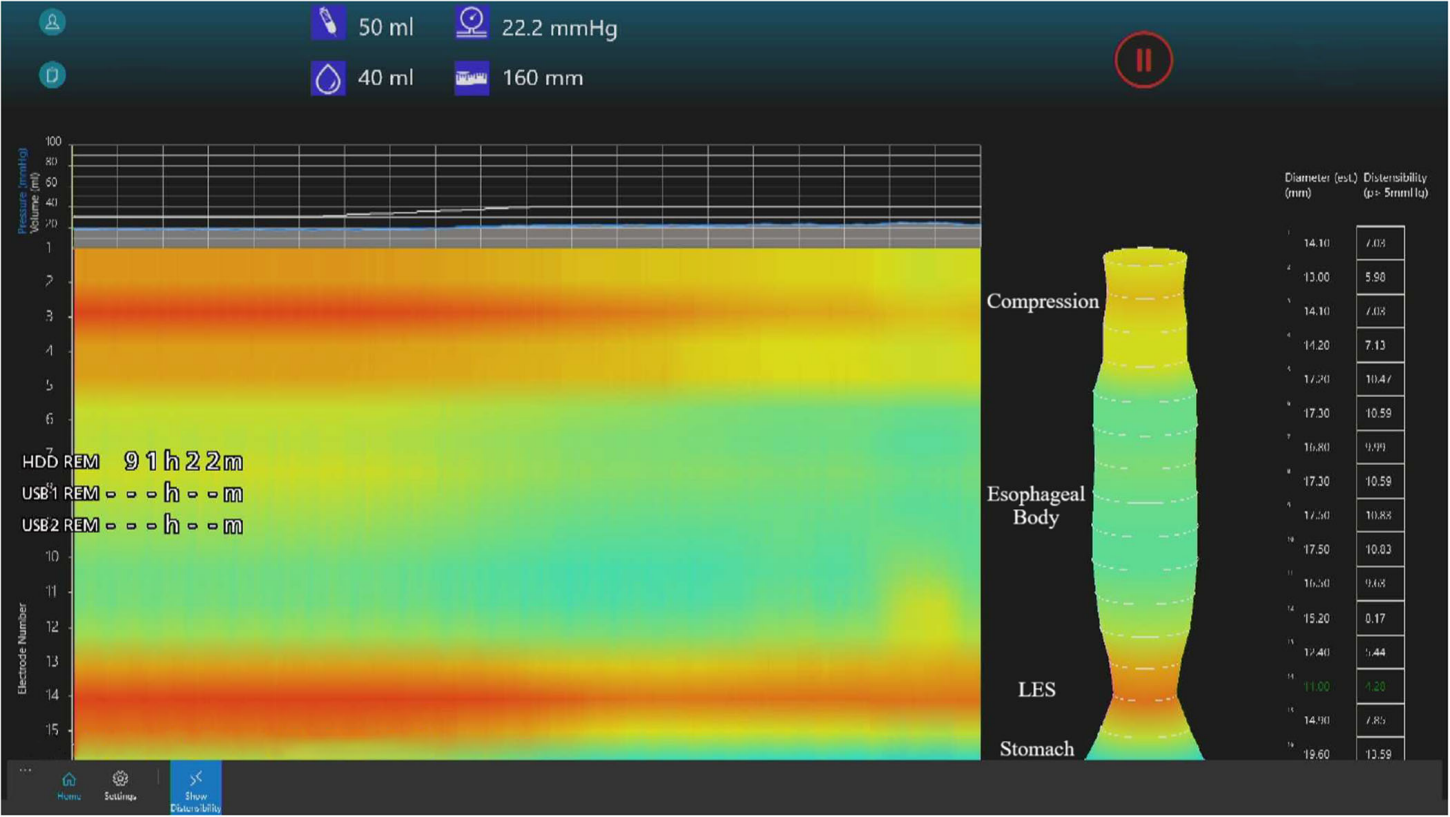
EndoFLIP topography showing persistent mid‐esophageal narrowing (Patient 1). Measurements at 40 mL balloon inflation reveal a reduced diameter and distensibility index (DI), consistent with a fixed narrowing at the site of compression. EndoFLIP, endoluminal functional lumen imaging probe; LES, lower esophageal sphincter.

Representative examples are provided in Figure 1 from Patient 1, showing persistent narrowing in the mid‐esophagus with reduced diameter and DI on EndoFLIP, and Supporting Information: Video 1 from Patient 4, showing pulsatile compression of the upper esophagus during endoscopy.

### 3.3 Paired LES versus compression site measurements

Two patients had matched‐volume measurements comparing the LES with the site of narrowing. For Patient 3 (30–40 mL balloon volume), the LES diameter ranged from 14.0 to 20.0 mm with a DI of 9.0–10.0, whereas the compression site measured 4.8–7.5 mm with a DI of 1.2–1.7. For Patient 4, the LES diameter was 20.0 mm with a DI of 6.0, compared to a compression site diameter of 10.3 mm and DI of 2.4, using representative 30 mL balloon volume measurements for direct comparison. These findings demonstrate reduced diameter and DI at the compression site in both patients. Although statistical testing could not be performed because of the small sample, the direction and magnitude of change in diameter and DI between the LES and compression site were consistent across both patients, supporting physiologic relevance of EndoFLIP findings. Compression‐site EndoFLIP data were not applicable in 2/8 patients (Patients 2 and 6; no EndoFLIP‐defined narrowing) and were not obtained in one patient with narrowing (Patient 8).

### 3.4 Management and outcomes

Treatments included surgical repair (*n* = 2 performed; *n* = 1 planned), dietary modification (*n* = 2), and proton‐pump inhibitor (PPI) therapy (*n* = 3). “Resolution” was defined as complete absence of dysphagia or feeding difficulty at follow‐up, while “partial improvement” denoted symptom reduction without full resolution, based on chart documentation. Objective dysphagia scoring was not available due to the retrospective design. One patient who underwent surgical repair of DAA experienced full symptom resolution, while two had partial improvement. One of these (Patient 1) underwent ARSA reimplantation with EndoFLIP assessment before and after surgery, which demonstrated no change in diameter or DI at the compression site (pre: 10.0–11.0 mm, DI 4.2–5.0; post: 10.0–11.0 mm, DI 3.7–4.5) and persistently diminished contractility of unclear etiology.

PPI therapy resolved symptoms in all three treated patients (Patients 2, 6, and 8), none of whom had significant functional obstruction on EndoFLIP. In all three patients, CT angiography demonstrated an ARSA; mild esophageal indentation was seen in Patients 2 and 8, whereas no definite compression was evident in Patient 6. In Patients 2 and 6, who had no narrowing on endoscopy or EndoFLIP, symptom resolution with PPI therapy was noted. Patient 8 had mild luminal narrowing on both endoscopy and EndoFLIP, with biopsy‐confirmed eosinophilic esophagitis. Complete resolution of symptoms on PPI therapy was observed in this case as well.

## 4 DISCUSSION

In this small heterogeneous pediatric cohort, EndoFLIP was safe, feasible, and provided complementary physiologic information that helped clarify the functional significance of vascular anomalies causing suspected esophageal compression. In several cases, EndoFLIP identified subtle narrowing not appreciated on endoscopy, while in others, it helped exclude meaningful functional obstruction despite radiographic impressions of indentation. Narrowing was identified more often with EndoFLIP than with endoscopy (75% vs. 50%), and in two patients, narrowing was detected despite normal endoscopic appearance. These preliminary observations suggest that EndoFLIP may offer additional insight when traditional modalities provide discordant or equivocal results, although the small sample size and retrospective design limit the strength of these findings.

These observations align with known limitations of visual endoscopic estimation. Prior pediatric work by Yasuda et al. has demonstrated substantial interobserver variability in “eyeball” assessments of esophageal caliber,^10^ and multiple adult studies similarly show frequent inaccuracies when estimating polyp size and subtle strictures.^11^, ^12^, ^13^ Recent American College of Gastroenterology (ACG) guidelines further highlight these limitations, noting poor sensitivity of endoscopic visual estimation in both children and adults and frequent discordance between endoscopic impressions and objective measurements like impedance planimetry.^13^ These well‐described limitations illustrate the potential value of objective measurements such as those provided by EndoFLIP.

In our cohort, these principles were reflected in three patients whose symptoms resolved with PPI therapy. In these cases, EndoFLIP demonstrated no significant functional narrowing (Patients 2 and 6) or only very mild narrowing (Patient 8), supporting non‐vascular causes of dysphagia despite the presence of vascular anomalies. CT angiography confirmed an ARSA in all three, showing mild esophageal indentation in Patients 2 and 8 and no definite compression in Patient 6. In Patients 2 and 6, UGI studies suggested narrowing despite normal endoscopy and EndoFLIP findings, reinforcing that radiographic impressions may not always reflect functional significance. Patient 8 had biopsy‐confirmed eosinophilic esophagitis, and complete symptom resolution with PPI therapy further supported an inflammatory rather than vascular etiology. Collectively, these cases illustrate how EndoFLIP can complement cross‐sectional or fluoroscopic imaging by providing real‐time functional assessment. This approach may help determine whether apparent vascular compression truly produces luminal restriction or represents an incidental anatomic impression, thereby reducing unnecessary surgical intervention.

In the subset of patients with paired measurements, diameter and DI were consistently lower at the compression site compared with the LES, suggesting that EndoFLIP may help quantify the relative severity of narrowing. In a related example, Patient 7, assessed only after surgical repair, demonstrated a normal DI at the prior compression site, coinciding with partial symptom improvement. Although the utility of DI at the LES has been well described, our findings provide preliminary evidence that assessment at extrinsic compression sites may also offer complementary diagnostic value. Distensibility in the mid‐esophageal body is typically higher than at the LES, which has implications when comparing DI values between the compression site and LES.^14^ These differences should be interpreted within this physiologic context rather than as absolute pathologic thresholds.

Interpretation of EndoFLIP findings in pediatrics remains challenging due to the absence of validated normative reference values by age and size. Balloon volume selection and catheter length must be individualized, as younger children cannot tolerate adult‐sized volumes, and smaller distension ranges may limit comparability across cases. Prior studies have shown that DI and diameter scale with body weight and esophageal length, emphasizing the need for standardized, age‐specific reference ranges.^6^, ^15^, ^16^ Studies in healthy adults report median LES DI values of approximately 3–9 mm^2^/mmHg.^17^

Published experience with EndoFLIP in vascular compression is limited and consists mainly of isolated case reports. In pediatrics, a recent report described intraoperative EndoFLIP use during repair of a right aortic arch with ALSA, Kommerell′s diverticulum, and left ligamentum arteriosum. The procedure showed immediate improvement in luminal caliber and impedance, with durable symptom resolution.^9^ In adults, EndoFLIP has been applied in the evaluation of suspected dysphagia lusoria. In one case, symptoms initially attributed to vascular compression were ultimately found to be due to achalasia. EndoFLIP demonstrated severely reduced distensibility and absent peristalsis at the LES, prompting successful pneumatic dilation.^18^ Another case demonstrated EndoFLIP detection of mid‐esophageal narrowing from an ARSA in an adult with non‐specific dysphagia and inconclusive initial testing. These findings were later confirmed by CT and symptoms resolved after hybrid vascular repair.^3^ These reports highlight both the potential of EndoFLIP to provide objective, real‐time functional assessment in operative and diagnostic contexts, and the importance of recognizing that vascular anomalies may be incidental or co‐exist with other esophageal disorders. Our series builds on these observations by examining multiple pediatric vascular anomaly types and comparing EndoFLIP findings with endoscopic and radiographic assessments, including one case evaluated after surgical repair.

Our findings suggest that EndoFLIP may help identify subclinical restriction not evident on endoscopy, provide objective measures of narrowing severity, and provide insight into functional status after surgical repair. However, the small, heterogeneous sample and retrospective design limit the strength of these conclusions. Variability in balloon volumes, sedation techniques, and incomplete or missing site‐specific data further reflect the current use of EndoFLIP in pediatric clinical practice, where standardized protocols are lacking. Prospective, multicenter studies with protocolized measurements and age‐specific normative reference data are needed to clarify how EndoFLIP parameters correlate with symptoms, predict surgical outcomes, and guide patient selection for intervention.

## 5 CONCLUSION

EndoFLIP offers real‐time quantitative assessment of luminal diameter and distensibility that may complement imaging and endoscopy in the evaluation of pediatric vascular esophageal compression. Although preliminary, our findings support further investigation into its potential role as part of a multimodal diagnostic and follow‐up strategy.

## CONFLICT OF INTEREST STATEMENT

Brett J. Hoskins: Consultantship: Mirum Pharmaceuticals, Inc. and 3‐D Matrix, Inc. Research Support: Travere Therapeutics, Inc. and Mirum Pharmaceuticals, Inc. Paroma Bose and Ryan T. Pitman. All other authors have no conflict of interest.

## Supporting information


**Supplemental Video 1**. Endoscopic view demonstrating pulsatile compression in the upper esophagus (Patient 4). The rhythmic indentation corresponds to vascular compression from the aberrant vessel.
